# Synchrotron X-rays reveal the modes of Fe binding and trace metal storage in the brown algae *Laminaria digitata* and *Ectocarpus siliculosus*

**DOI:** 10.1093/mtomcs/mfad058

**Published:** 2023-09-22

**Authors:** Ana Mijovilovich, Peter Cloetens, Antonio Lanzirotti, Matt Newville, Gerd Wellenreuther, Puja Kumari, Christos Katsaros, Carl J Carrano, Hendrik Küpper, Frithjof C Küpper

**Affiliations:** Czech Academy of Sciences, Biology Centre, Institute of Plant Molecular Biology, Laboratory of Plant Biophysics and Biochemistry, Branišovska 1160/31, 370 05 Česke Budějovice, Czech Republic; ESRF—The European Synchrotron Radiation Facility, Beamline ID16A, 71, avenue des Martyrs CS 40220 38043 Grenoble Cedex 9, France; Argonne National Laboratory, The University of Chicago, Building 434A, 9700 South Cass Avenue, Lemont, IL 60439, USA; Argonne National Laboratory, The University of Chicago, Building 434A, 9700 South Cass Avenue, Lemont, IL 60439, USA; European XFEL GmbH, Holzkoppel 4, 22869 Schenefeld, Germany; School of Biological Sciences, University of Aberdeen, Cruickshank Building, St Machar Drive, Aberdeen AB24 3UU, UK; Department of Biology, National and Kapodistrian University of Athens, Panepistimiopolis, Athens 157 84, Hellas, Greece; Department of Chemistry and Biochemistry, San Diego State University, CA 92182-1030, USA; Czech Academy of Sciences, Biology Centre, Institute of Plant Molecular Biology, Laboratory of Plant Biophysics and Biochemistry, Branišovska 1160/31, 370 05 Česke Budějovice, Czech Republic; Department of Experimental Plant Biology, University of South Bohemia, Branišovská 31/1160, 370 05 České Budějovice, Czech Republic; School of Biological Sciences, University of Aberdeen, Cruickshank Building, St Machar Drive, Aberdeen AB24 3UU, UK; Department of Chemistry and Biochemistry, San Diego State University, CA 92182-1030, USA; Marine Biodiscovery Centre, Department of Chemistry, University of Aberdeen, Aberdeen AB24 3UE, UK

**Keywords:** algae, iron, strontium, tomography, XANES, ferritin

## Abstract

Iron is accumulated symplastically in kelp in a non-ferritin core that seems to be a general feature of brown algae. Microprobe studies show that Fe binding depends on tissue type.

The sea is generally an iron-poor environment and brown algae were recognized in recent years for having a unique, ferritin-free iron storage system. Kelp (*Laminaria digitata*) and the filamentous brown alga *Ectocarpus siliculosus* were investigated using X-ray microprobe imaging and nanoprobe X-ray fluorescence tomography to explore the localization of iron, arsenic, strontium, and zinc, and micro-X-ray absorption near-edge structure (μXANES) to study Fe binding. Fe distribution in frozen hydrated environmental samples of both algae shows higher accumulation in the cortex with symplastic subcellular localization. This should be seen in the context of recent ultrastructural insight by cryofixation–freeze substitution that found a new type of cisternae that may have a storage function but differs from the apoplastic Fe accumulation found by conventional chemical fixation. Zn distribution co-localizes with Fe in *E. siliculosus*, whereas it is chiefly located in the *L. digitata* medulla, which is similar to As and Sr. Both As and Sr are mostly found at the cell wall of both algae. XANES spectra indicate that Fe in *L. digitata* is stored in a mineral non-ferritin core, due to the lack of ferritin-encoding genes. We show that the *L. digitata* cortex contains mostly a ferritin-like mineral, while the meristoderm may include an additional component.

## Graphical Abstract

**Graphical Abstract:**
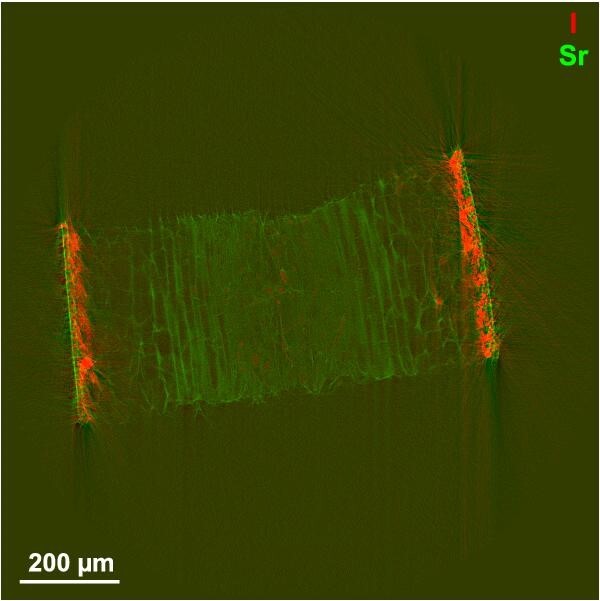
Distribution of Strontium in a cross-section of a thallus of the brown alga (kelp) *Laminaria digitata*, visualized by nanoprobe XRF tomography.

## Introduction

The sea is generally an iron-poor environment, and marine life has evolved a plethora of unique adaptations to cope with this situation—chiefly by either having high-affinity uptake and recycling systems for the little iron that is available or using chemical alternatives to iron.^1^ Iron is essential for all living organisms due to its ubiquitous role in redox and other enzymes, especially those involved in respiration and photosynthesis. The iron uptake and storage systems of terrestrial plants are now reasonably well understood. Ferritin represents the most common form of iron storage in all domains of life.^2–4^

Most heterokont organisms are an exception to ferritin storage. They have developed as an independent lineage of plant-like organisms during the crown diversification of eukaryotes, which makes them particularly interesting organisms for studying physiological and biochemical adaptations.^5,6^ An iron storage system without ferritin was initially recognized in a centric diatom^7^ and then confirmed in the first brown algal genome project of the filamentous *Ectocarpus siliculosus*.^5,8^ This feature is shared with other heterokont organisms such as oomycetes.^9^ Instead, brown algae use iron–sulfur clusters as iron stores intracellularly.^10^ Another two species lacking ferritin have been found among haptophytes (*Emiliania huxleyi* and *Pavlovales* sp.) in an *in silico* study.^11^ For the species considered in that study, chlorophytes, cryptophytes, and several species of the SAR supergroup (Stramenopiles, Alveolates, and Rhizarians) use ferritin to accumulate iron. A lot of knowledge on Fe uptake and storage in algae is still based on *in silico* studies of protein sequences but less has been done using biochemical and biophysical characterization of Fe uptake and storage.^11^ An amorphous polymeric iron-oxo/phosphato mineral core similar to ferritin cores but a lot larger than normal bacterial ferritins was found in *E. huxleyi* by X-ray absorption spectroscopy (XAS) and Mössbauer spectroscopy. *Emiliania huxleyi* lacks ferritin-encoding genes. The study lacked cellular resolution to distinguish whether Fe was stored in a ferritin-like protein or in the vacuole.^12^

Even less is known about mechanisms and ultrastructure of the accumulation of zinc, strontium, and arsenic in brown algae. In all of these cases, understanding of function has been limited by lack of knowledge of storage locations at the subcellular level.

Advances in X-ray synchrotron nano- and micro-probes allow studies of the metal distribution of frozen hydrated samples at the tissue and cell levels. X-ray probes map all the metals in the tissue, different from fluorescent protein tags or other labels that are restricted to the labelled metal subset, and usually also limited to a particular redox state of that metal. Further, while all probes that visualize metals by binding to them can only report metals that are weakly enough bound so that the probe can successfully compete with the other ligands in the cell, imaging based on X-ray emission (Energy Dispersive X-ray Spectroscopy [EDX], Particle-induced X-ray Emission [PIXE], and X-ray Fluorescence [XRF]) detects all metals regardless of their ligands and redox state. Nowadays, chemical speciation with XAS is also possible at micrometre resolution in confocal or tomography mode.^13,14^ Micro- and nanoprobes can show *in situ* whether Fe is accumulated in the apoplast or in a vacuole (active accumulation by transmembrane transport proteins like ATPases, CDFs, or NRAMPs).

Cell wall binding is always controversial when samples are prepared by fixation or freeze drying. Chemical fixation can wash out the cell liquid content though the cell wall, leading to re-location of metals. Plunge freezing followed by freeze drying is better than chemical fixation but still can lead to a distorted elemental composition.^15^ Cell walls can passively adsorb trace metals, so that they are even used as ion-exchange resins to unspecifically remove metals from wastewater.^16^ Due to this feature of cell walls, re-location of dissolved metals in improperly prepared samples for metal imaging is not uncommon (e.g. in micro-X-ray fluorescence [μXRF], EDX, PIXE, and Nanoscale Secondary Ion Mass Spectrometry [nanoSIMS]). Scanning nanodiffraction experiments of living and fixed cells have shown that even nanostructures can be formed by the fixation procedure.^17^ This artefact is avoided in intact, shock-frozen hydrated samples, if possible measured in tomography mode in synchrotron micro- and nanoprobes.

Seminal papers in 2012 determined an Fe atypical storage in several algae species and have set the standard for the methodology for the last decade with the use of various spectroscopies and microscopy.^10,12,18^ Nowadays, it is possible to directly measure the metal distribution, and the chemical speciation by X-ray absorption near-edge structure (XANES) with spatial resolution, due to recent development of very brilliant micro- and nanoprobes at synchrotrons. This work introduces a change in the methodology to study metal distribution in kelp incorporating the recent developments in photon science and intact frozen sample preparation, with new findings and some revisited questions.

In this study, we investigated the distribution of Fe, As, Zn, Sr, and K in environmental samples of the kelp *L. digitata* with a spatial resolution in the hundreds of nanometres that allows attaining cellular resolution. The metal distributions in cultured *E. siliculosus* were also measured. Previous studies of iron binding in *E. siliculosus* were done for entire algal thalli, giving an average of the binding over all tissues.^10^ In the present study, we investigated Fe binding by μXANES of the Fe K-edge with tissue resolution on frozen hydrated tissues of *L. digitata.*

This paper shows that at cellular resolution in intact frozen hydrated samples measured in tomography mode, it is possible to unequivocally determine whether metals are accumulated at the cell wall or inside the cell, and that there are differences in the Fe binding between tissues (cortex and meristoderm). Spectroscopy together with genome information is used to determine the chemical form of the stored iron.

## Materials and methods

### Samples

For X-ray microprobe studies, *E. siliculosus* (CCAP 1310/4) was cultured at the Culture Collection of Algae and Protozoa (CCAP) in Oban in spring 2009. The same strain has previously served for sequencing the first macroalgal genome, but also significantly in the context of the present study in studies of iron and halogen metabolism.^5,8,10,19,20^

For tomography, *L. digitata* thalli were collected by snorkelling at Bullers of Buchan, Cruden Bay, Aberdeenshire, on 23 May 2017. Tomography samples were prepared by mounting them in polyimide capillaries, attaching them to sample holders, and shock-freezing the whole assembly in supercooled isopentane.^13,14^ Samples were stored in liquid N_2_ and transported in a dry shipper to the European Synchrotron (ESRF), Grenoble, France.

For scanning X-ray fluorescence and μXANES samples, *L. digitata* thalli were collected by snorkelling at Bullers of Buchan, Cruden Bay, Aberdeenshire, in October 2019. Samples were cut into small sections using a razor blade and subsequently frozen in a cryomould containing OCT (optimum cutting temperature compound, tissue freezing medium; VWR, Avantor, Lutterworth, Leicestershire, England). They were frozen in a cryostat (Leica CM1850 UV, Leica, Germany) using the Peltier element, which cools the quick-freeze station. The cryostat was set to −20°C and transverse sections were cut at 10–30 μm with the microtome. Sections were collected onto a Kapton film with another sheet of film placed on top to sandwich the section. They were mounted into sample holders using a cryostat (Leitz, Germany). This work was carried out at the Microscopy & Histology Facility of the Institute of Medical Sciences, University of Aberdeen. Samples were transported in dry ice to the Advanced Photon Source (APS), Argonne National Laboratory, IL, USA.

## Transmission electron microscopy

Transmission electron microscopy (TEM) images were produced with *L. digitata* thalli prepared by both conventional chemical fixation (CCF) and cryofixation-freeze substitution (CF-FS) as described in the literature.^21^

### Bioinformatic analysis of ferritin gene in brown macroalgae

A search was performed to identify putative ferritin genes of brown seaweeds in the National Center for Biotechnology Information (NCBI) genomic and proteomic database. Additionally, the conserved domain of the ferritin-like superfamily of diiron proteins containing four-helix bundles (cl00264: ferritin-like superfamily) was searched in the available genomic data of brown seaweeds (Supplementary Table S2).

### Single slice X-ray fluorescence tomography

Measurements of X-ray fluorescence tomography followed previously established protocols for X-ray emission and absorption spectroscopy in shock-frozen hydrated samples to prevent element re-distribution.^13,14,22,23^ Samples were measured at the cryo X-ray nanoprobe of beamline ID16A (ESRF), with a beam size of 20 nm × 20 nm, which determined the thickness of the tomographic slice.^24^ XRF photons were detected with a six-element silicon drift detector with xMAP™ readout electronics (XIA LLC, Hayward, CA, USA). This configuration was not fast enough, however, to obtain enough counts for working with a step size similar to the beam size for these large samples; therefore, the step size (= lateral resolution of the measurement) was 800 nm. Samples were brought to the beamline in a dry shipper and measured at approximately −150°C. Single slice tomograms were measured with an excitation energy of 33 500 eV providing access to the K-edge of iodine (33 170 eV). The spectra were fitted with the PyMca libraries, and the tomograms were reconstructed with a regularized maximum-likelihood expectation-maximization algorithm using an in-house software implemented from the ASTRA Toolbox and SPIRE wrapper (https://github.com/pierrepaleo/spire).^25,26^ Multielement standards were prepared in the same way in the same capillaries as the samples for full correction of absorption effects and quantitation of the elemental concentration in molar concentration.^14^ Three-dimensional tomograms of the density distribution were obtained by propagation-based X-ray phase-contrast imaging.^27^ Single distance phase retrieval was achieved with the contrast transfer function using the ESRF in-house software programmed in GNU Octave (www.octave.org). Images of μXRF and phase tomograms were further processed (quantification, colour scales, and colour space) in the ‘Fiji’ version of ImageJ.^28^

### Scanning μXRF and Fe K-edge XANES

Scanning μXRF images and Fe K-edge XANES spectra were obtained at 13ID-E beamline at the APS, Argonne Νational Laboratory, USA. Care was taken to ensure samples remained well frozen during transport using dry ice. At the beamline, samples were mounted in a Peltier cold stage, built by GSECARS, for analysis. The cold stage consists of a Peltier thermoelectric module cooled by a circulating ethylene glycol-water mixture. Kapton® brand polyimide film (8 μm thick) was used to cover the sample (preventing the formation of frost on the surface). Sample temperatures during analysis are typically approximately −20.0°C.^29^ The storage ring was running with an energy of 7 GeV at 102 mA. The beamline X-ray source is a 36 mm period undulator and for these experiments an Si(111) double-crystal monochromator cooled with liquid N_2_ was used to select energies. Focusing is achieved by a set of Kirkpatrick–Baez focusing mirrors focusing to a spot of ∼2 × 2 μm^2^ on the sample. The flux at the sample was ∼0.5 × 10^8^ photons s^−1^ at the energy of Br K-edge (13 550 eV). Scanning μXRF images were collected for excitation energies of 7200 and 13 550 eV and a pixel size of 3 μm.

The elemental deconvolution was done with LARCH.^30^ Semi-quantitative concentrations were calculated using a standardless fundamentals parameters approach using the measured Ca signal of the highest concentrated sample (L1) as internal standard. Fluorescence images of Fe, As, and Zn distribution were also obtained. XANES was collected on selected spots of those images for the Fe K-edge of *L. digitata*. Six spectra were averaged for meristoderm, and three for cortex.

The energy scan range was (−90 to +250 eV) around the Fe–K absorption edge. The Fe energy was calibrated by setting the first derivative of the absorption edge for iron metal to a value of 7111.7 eV. Data background reduction was performed with ATHENA by fitting a pre-edge and post-edge spline and normalizing the atomic absorption to one.^31^

For the composition analysis of the Fe K-edge XANES, we re-used data from our former experiments or high-quality spectra from the literature. First, the Fe coordination in the *E. siliculosus* bulk sample CC47 was determined with Mössbauer spectroscopy and extended X-ray absorption fine structure (EXAFS) data obtained in a former study, which was used in this study as reference.^10^ In a second approach, we re-used the spectra of ferrihydrite and Fe(III) sulphate from Pattammattel *et al*., which was measured with the same monochromator type as in this work, having similar line broadening.^32^ Plots were done with Origin7 (OriginLab Corporation, Northampton, MA). Images were assembled with Affinity Designer [Serif (Europe) Ltd., 2020].

## Results

### Metal distribution in *Laminaria digitata* and *Ectocarpus siliculosus*

Preliminary measurements of metal distribution in *E. siliculosus* (see Supplementary Fig S1) performed in 2009 with a beam of 10 μm showed accumulation of Br, Ca, Cl, Cu, Fe, I, K, Ni, Sr, and Zn but no accumulation of Ga and Mn, while S was found just above background. A slight amount of iodine can be seen from the K line.

**Fig. 1:**
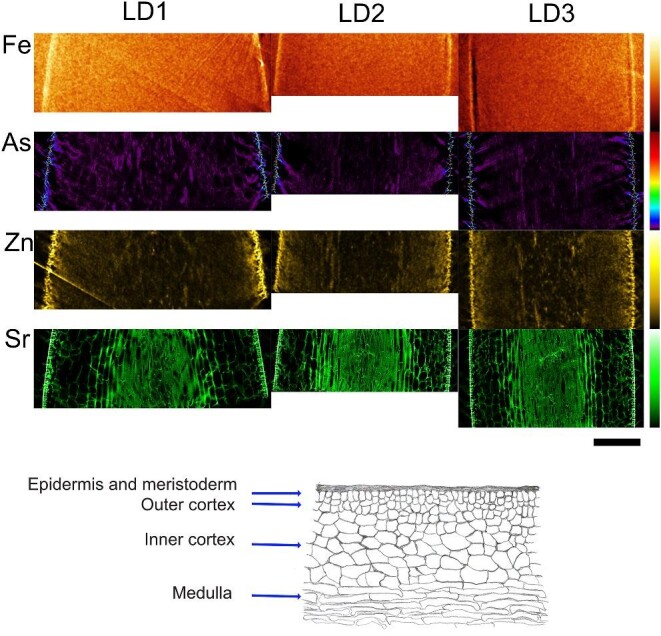
Single slice nano-X-ray fluorescence tomography of Fe, As, Zn, and Sr distribution in *L. digitata* (replicates LD1 and LD2). Colour bar scale in mg/kg (top is maximum of the scale): Fe [56–335]; As [0–749], Zn [0–98], and Sr [88–1314]. Scale bar: 150 μm. Bottom: sketch of brown algal tissues.

**Fig. 2:**
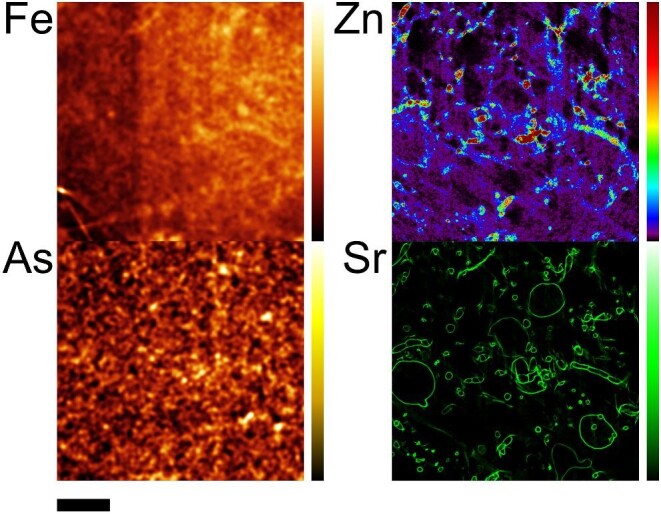
Single slice nano-X-ray fluorescence tomography of Fe, As, and Zn distribution in *E. siliculosus.* Colour bar scale in mg/kg (top is maximum of the scale): Fe [56–140]; As [1–4], Zn [0–118], and Sr [88–1314]. Scale bar: 150 μm.

Metal distribution was obtained by single slice nanotomography in intact frozen hydrated samples of both *L. digitata* (one blade) and *E. siliculosus* (several filaments caught in different positions in the single slice tomogram; Figs. 1 and 2). For *L. digitata*, the Fe fluorescence images do not show any cellular structure due to low metal concentration relative to the background and the low quantum yield of the high excitation energy of the beam (33 500 eV), which was optimized for iodine measurements on the same samples as part of a different study. In spite of those issues, the Fe distribution at the tissue level was obtained with most Fe appearing at the cortex. Fluorescence images of As and Zn also show the highest concentration at the cortex with both metals located inside the cells for all the tissues. In *E. siliculosus*, the Fe distribution was co-localized with Zn inside the cells. In contrast to this, As was localized in the cell wall of each filament, showing that the experimental conditions provide resolution to the subcellular level.

The nanotomographies show Sr localized in the apoplast (Fig. 1). The colocalization of Fe, Zn, and Sr (Supplementary Fig. S5) shows that Fe is located mostly in the cells, while Sr is located in the cell wall (green in the colocalization figure). X-ray contrasting phase tomography (collected just before the fluorescence tomogram) shows a high-density thin layer around the cell, shown in light grey (see Supplementary Fig. S7, zoomed image on the right). On the right side of Supplementary Fig. S7, the X-ray contrasting tomogram was manually aligned with fluorescence Sr nanotomography, with the dark areas showing the colocalization of high-density in the phase contrast image with the Sr high concentration of the fluorescence tomogram. The structural similarity index map (SSIM) compares luminance, contrast, and structure between the Fe/Zn and Sr (highest similarity at top of colour bar in red colour).^33^ SSIM shows colocalization of Fe and Sr in the medulla (see yellow areas at bottom left of panel), but Fe does not seem to colocalize in the cell walls, while Zn and Sr colocalize in almost all tissues of the blade and also at the cell walls (seen as yellow lines).

**Fig. 3:**
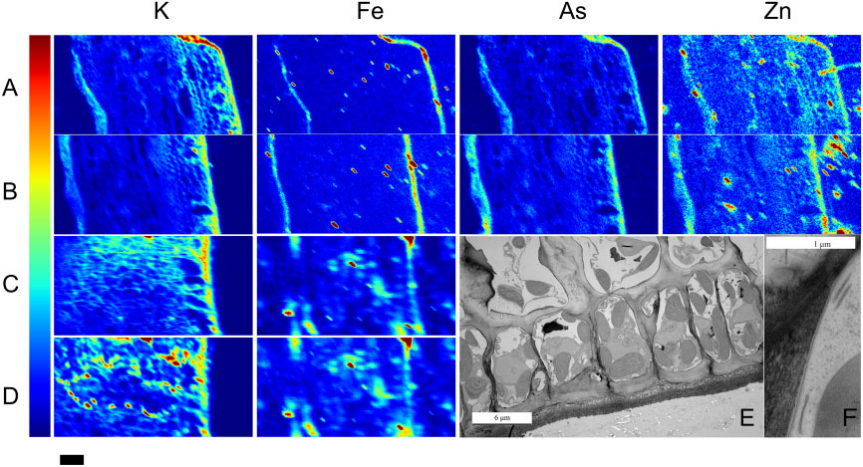
(A–B) Scanning micro-X-ray fluorescence (μXRF) images for K, Fe, As, and Zn in two samples of *L. digitata* collected with excitation energy of 13 550 eV (above the Br K-edge, experiment was optimized for bromine). Notice that the fluorescence yield for K is lower at this higher energy. (C–D) Scanning μXRF images for K and Fe in three samples of *L. digitata* collected with excitation energy of 7200 eV (close above the Fe K-edge) for best fluorescence yield. Scale bar 250 μm. Colour bar: red for highest concentration, intensity normalized to Ca signal. Representative cross section images of *L. digitata*: (E–F) high resolution (transmission electron microscopy, TEM); (E) tissue prepared by conventional chemical fixation (CCF; scale bar 6 μm; F) high magnification of a cell wall after cryofixation-freeze substitution (CF-FS; scale bar 1 μm; F).

Scanning μXRF images of Fe distribution in *L. digitata* were collected at two energies closer to the Fe K-edge, resulting in higher quantum yield. In these fluorescence images, Fe is found mainly in the cortex (see Fig. 3). The concentration in the internal tissues is at the level of the background signal, which is below detection limit. This is different from Zn and As, which show higher concentrations in the cortex as well but a concentration above background in the internal tissues. The images collected with an excitation energy of 7200 eV (see Fig. 3C and D), close to the Fe K-edge, show more structure in the internal distribution than those collected at 13 550 eV (in a beamtime optimized for bromine; see Fig. 3A and B) due to the decreased fluorescence yield at higher energies. In *L. digitata*, Sr appears with high intensity and bound to the cell wall in the cortex tissue, but there is a homogeneous distribution in the meristoderm. Figure 3E is a cross thallus section of a representative sample showing epidermal cells after CCF, and 3F shows numerous flat cisternae (term introduced by Katsaros *et al*. 2021) parallel to a cell wall after CF-FS.^21^ These structures are not visible after CCF.

In the nanotomography of *E. siliculosus* (see Fig. 2 and the metal colocalization in Supplementary Fig. S6), Sr was found in the cell wall. The SSIM for *E. siliculosus* shows more colocalization with Sr for Zn than for Fe (see Supplementary Fig. S6)^33^. The concentration of As in *E. siliculosus* is much lower than in *L. digitata*, but the low signal to noise ratio hampers further interpretation.

### Fe binding in *L. digitata* tissues

**Fig. 4:**
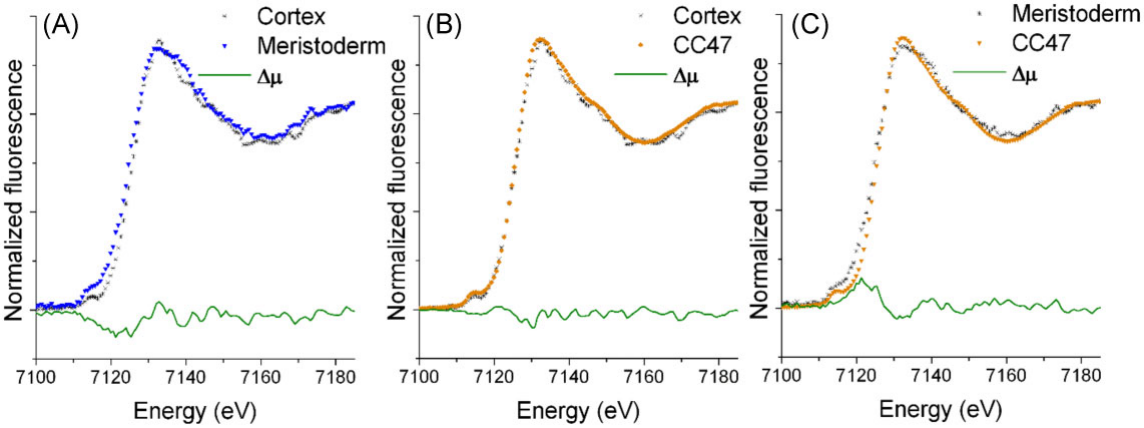
Fe K-edge micro-X-ray absorption near-edge structure (XANES) of (A) cortex and meristoderm of *L. digitata*, and comparison of the *E. siliculosus* bulk measurement (CC47) of Böttger *et al*. (2012) with (B) *L. digitata* cortex and (C) meristoderm. Difference spectra (Δμ) are shown for each case.

Fe K-edge XANES collected on selected spots of the micro-fluorescence images of *L. digitata* are shown in Fig. 4A. The spectra show a reasonable statistical quality for this type of experiment (microprobe of single phylloid blade) in a non-accumulator organism, as we have found also in the non-accumulator land plant *Noccaea ochroleucum*.^13^ Spectra of the meristoderm and cortex have a similar edge energy position, indicating no difference in oxidation state but maybe a difference in composition. The tiny difference in intensity but not in shape of the pre-edge feature at about 7112 eV between cortex and meristoderm needs further investigation, since differences in data quality, due the higher concentration of Fe in the cortex than in the meristoderm can affect the fitting of the pre-edge spline in the data reduction of the lowest Fe concentration sample. Spectral shape is not affected by data reduction, and differences in the spectra are found in the lower energy side of the rising edge for the meristoderm sample (small shoulder) and the wider peak apex of the main resonance, respect to the spectrum of the cortex.

The Fe K-edge XANES of *E. siliculosus* that was characterized by Mössbauer spectroscopy and EXAFS collected on a whole blade (bulk sample) measured in a beamline for bulk EXAFS (non-microprobe) was used as reference.^10^ Figure 4B and C shows that XANES of the cortex for *L. digitata* is the same as in *E. siliculosus*, while there are slight differences between the meristoderm of *L. digitata* and *E. siliculosus*.

The difference analysis (called Δμ) is more revealing of these tiny differences.^34,35^ Figure 4A–C shows Δμ of the micro-probe cortex and meristoderm in *L. digitata* compared to the bulk spectrum of *E. siliculosus* (sample CC47), shows differences in the rising edge and peak apex.

A linear combination fit shows that the cortex of *L. digitata* has 98% similarity to the spectrum of the whole blade of *E. siliculosus*, while only 72% of the *L. digitata* meristoderm can be explained by the *E. siliculosus* spectrum (see further details in the Supplementary Fig. S2 and Table S1).

The linear combination fit of the bulk samples of *E. siliculosus* and the *L. digitata* cortex and meristoderm, using ferrihydrite and Fe(III) sulphate from the literature, can only be fitted with ferrihydrite, giving a bad quality fit (see Supplementary Figs. S3 and S4) that seems missing a component different from Fe(III) sulphate.^32^

### Bioinformatic analysis of ferritin in brown seaweeds

The search with keywords ‘ferritin and algae’ gave a total of 152 hits in NCBI (accessed on 22 October 2022), of which 4 belong to brown seaweeds, namely *Fucus vesiculosus, Sargassum fusiforme, Sargassum horneri*, and *Sargassum thunbergii* (Supplementary File S1). The gene was annotated as *acs*F (aerobic cyclase system Fe-containing subunits). The search for the conserved domain of the ferritin-like superfamily (cl00264) also suggested the presence of *acs*F genes in 29 brown seaweeds including *E. siliculosus* and *L. digitata* (Supplementary Table S2). *acs*F has a ferritin-like diiron-binding domain, and it is located in the chloroplast. The *acs*F gene is found in a wide range of photosynthetic organisms and is primarily involved in aerobic oxidative cyclization of Mg-protoporphyrin IX monomethyl ester.

However, no ferritin genes or protein were identified in the genome and identified protein database of any seaweeds (listed in Supplementary Table S2) containing either of the conserved domains Ferritin-like (cd00657), Ferritin (cd00904), or Nonheme_Ferritin (cd01055) responsible for iron storage, which are the members of the same broad superfamily of ferritin-like diiron carboxylase proteins.

## Discussion

Images of the intact sample in tomography as well as the micro-fluorescence images of the sample slices show a low Fe concentration. Zn distribution maps obtained by both tomography and 2D scanning methods show zones devoid of the metal. These depleted zones cannot be attributed to sample damage during cutting or thawing at transport, since the tomography done on intact frozen tissue shows the same voids.

For the nanoprobe tomography on intact frozen hydrated tissues, the high excitation energy lowered the quantum yield for Fe and the images lack intracellular resolution in *L. digitata*. The microprobe with an excitation energy closest to the Fe K-edge, measuring on slices cut from frozen hydrated tissues, shows more structure of the internal tissues though not enough for cellular resolution in *L. digitata*. Both probes agree on the highest Fe accumulation in the cortex and a rather homogeneous accumulation in the inner tissues of *L. digitata.* In this context, the role of the new type of cisternae-like structure (so-called flat cisternae) discovered by a new sample preparation method by CF-FS in recent ultrastructure microscopy images of *L. digitata*, which have an electron-dense content, should be investigated for a potential role in metal and halide storage.^21^ These structures are not visible in sections of material prepared by conventional CCF. They are clearly visible after CF-FS, and usually appear close to cell walls, with particularly high numbers parallel to primary pit fields. It was suggested that they could function for transport and alternative storage to the vacuole. Our study is in agreement with Katsaros *et al*.,^21^ but the resolution of our tomography study does not allow to see the cisternae, which are clearly visible after CF-FS.^21^

Sr binds strongly to alginate and turned out to be ideally suitable for imaging the cell walls with μXRF. In *L. digitata*, Sr shows accumulation in the cell wall in the cortex tissue, while the meristoderm shows a rather homogeneous distribution.^36^ The X-ray phase-contrasting tomograms together with the Sr fluorescence tomograms locate Sr in a thin layer, consistent with Sr accumulation in the cell wall. Comparison with TEM (see Fig. 3F and G) shows that Sr can be used as proxy for the cell wall and to reveal the structure of the tissue. A similar distribution is found for As. A nanoSIMS study on fixed samples imaging a few cells detected As in the cell wall and in the apoplast, with less in the cell membrane.^37^ At first glance, this suggests an efficient As detox system of the cytosol and other subcellular tissues. However, as chemical fixation is known to be able to relocate mobile ions in tissues, and the cell wall can act as unspecific ion-exchange resin, this result would need a verification with other preparation methods.^16^ Our study of one tomographic slice shows that in the meristoderm As (and also Sr) has a homogeneous distribution, suggesting a different mechanism of dealing with As for the different tissues, or just a strong dependence of the As concentration on the parameters governing the solubility in sea water (like salinity).

It has been reported for *E. siliculosus*, based on chemical staining and EDX on chemically fixed samples, that Fe would accumulate in the cell wall.^20^ Newly prepared *L. digitata* samples prepared by CCF agree with former studies on *E. siliculosus* in showing accumulation at the cell wall, and do not show the cisternae, which are only visible when prepared by CF-FS.^20,21^ X-ray fluorescence tomography of an intact frozen hydrated sample of this work shows Fe co-localized with Zn, and the Fe is distributed in all the filament, predominantly inside the cells, as seen at the resolution of this experiment (20 nm beam size = thickness of the tomographic slice, but 800 nm step size = lateral resolution). The Sr image provides the location of the cell wall, but the lower As concentration does not provide subcellular resolution.

The genome of brown algae lacks the genes to encode ferritin. Therefore, a different mode of Fe storage can be expected for brown algae. The mineral core resembling ferritin found in *E. siliculosus* was assigned to an amorphous mineral similar to the ferrihydrite core of ferritin, according to the results of Mössbauer spectroscopy and XAS.^10^ The Fe K-edge XANES of *L. digitata* of our study is very similar to those of *E. siliculosus* and a ferritin-like mineral core. The *L. digitata* chloroplast genome (https://www.ncbi.nlm.nih.gov/bioproject/PRJNA573202) lacks ferritin-encoding genes similar to *E. siliculosus. Laminaria digitata* along with *Laminaria rodriguezii* and *Laminaria solidungula* contains genes for acsF phytochrome regulated protein and *E. siliculosus* contains acsF magnesium–protoporphyrin IX monomethyl ester (oxidative) cyclase, all belonging to the yc59 protein family (CHL00185) of the ferritin-like diiron carboxylase superfamily conserved domain. These findings suggested that the non-ferritin mode of iron storage is present in the cortex of the *L. digitata* of this work.

The tissue resolution provided by the microprobe showed that there is a slight difference between Fe binding in the meristoderm and the cortex, which needs further investigation with more effort in studying the meristoderm containing very low Fe levels. Mössbauer spectroscopy has shown that the Fe mineral has a bigger size than bacterial or plant ferritins, which may indicate a higher capacity of Fe storage. The *L. digitata* cortex of this study seems very similar to that of *E. siliculosus*, which was determined to be mostly a ferrihydrite mineral (74%) and about 26% of an Fe–S cluster.^20^ The fit of the whole tissue *E. siliculosus* XANES region with *L. digitata* cortex (72%) and meristoderm (28%) gives a composition that suggests a greater association of the ferrihydrite mineral with the *L. digitata* cortex, while the meristoderm seems to have a higher concentration of another component.

To check the plausibility of the hypothesis, we performed fits with ferrihydrite and Fe(III) sulphate as components. The fit of the μ-XANES of the cortex and the meristoderm, using the bulk sample of *E. siliculosus* (sample CC47), gives a better fit than using ferrihydrite and Fe(III) sulphate as components. This means that the Fe–S cluster found with EXAFS in the *E. siliculosus* sample cannot be reproduced by Fe(III) sulphate. Visual inspection of the fits shows that besides ferrihydrite another minor component is missing.

Previous studies using Mössbauer spectroscopy and EXAFS have paved the way for the current spectroscopy studies with spatial resolution. However, Mössbauer spectroscopy studies require enrichment with the ^57^Fe isotope, requiring several weeks of treatment, which hampers measuring fresh environmental samples. Microprobes can provide spectroscopic data on fresh natural samples, and at best in tomography mode, avoiding sample damage during slicing.

In summary, the present work using synchrotron micro- and nanoprobes advances the knowledge about metal distribution in *L. digitata* and *E. siliculosus* to the tissue and cellular levels. We have shown that in *L. digitata*, all metals are concentrated in the cortex. In the meristoderm and medulla, Fe has a homogeneous distribution in *L. digitata*, and is found inside the cells in *E. siliculosus*, different from the cell wall accumulation found from staining micrographs.^20^ The nanoprobe tomography of this work and the current sample preparation by CF–FS for ultrastructures show that intact frozen samples are necessary to study kelp at the tissue and subcellular levels. Sr and As co-localized with As in the medulla. The different distribution of As, Sr, and Zn in different tissues points to different processes for metal uptake and accumulation. Fe accumulates in *L. digitata* as a non-ferritin ferrihydrite mineral mostly at the cortex and with a lower content in the meristoderm.

The results of this work on environmental kelp samples using X-ray micro- and nanoprobes suggest that the formation of an amorphous mineral might be the storage strategy of non-ferritin-containing algae. The findings of this work suggest that the modes of accumulation and Fe binding are different depending on tissue type. At difference from former work with different sample preparation, we showed that Fe accumulation occurs inside the cell and that Sr is located in the cell wall.

## Supplementary Material

mfad058_Supplemental_File

## Data Availability

Data are available upon reasonable request to the corresponding author. Raw data collected at ESRF is available from data portal. Kuepper, F. C., Kuepper, H., & Mijovilovich, A. E. (2021). Exploring the localization and potential functional links between stored iodine and iron in brown algae [dataset]. European Synchrotron Radiation Facility. https://doi.org/10.15151/ESRF-ES-102373644.
