# Supplementary material for: Synchrotron X-rays reveal the modes of Fe binding and trace metal storage in the brown algae Laminaria digitata and Ectocarpus siliculosus
Source: Metallomics. 2023 Sep 22;15(10):mfad058. doi: 10.1093/mtomcs/mfad058 (PMC10588612; doi:10.1093/mtomcs/mfad058)
Supplement: mfad058_Supplemental_File [file mfad058_supplemental_file.docx]

**Supplementary information**

**Synchrotron X-rays reveal the modes of Fe binding and trace metal storage in the brown algae Laminaria digitata and Ectocarpus siliculosus.**

Ana Mijovilovich^1^, Peter Cloetens^2^, Antonio Lanzirotti^3^, Matt Newville^3^, Gerd Wellenreuther^4^, Puja Kumari^5^, Christos Katsaros^6^, Carl J. Carrano^7+^, Hendrik Küpper^1,8^, and Frithjof C. Küpper^5,7,9#^

^1^ Czech Academy of Sciences, Biology Centre, Institute of Plant Molecular Biology, Laboratory of Plant Biophysics and Biochemistry, Branišovská 31/1160, 370 05 České Budějovice, Czech Republic.

^2^ ESRF – The European Synchrotron, Beamline ID16A, Grenoble, France.

^3^ The University of Chicago, Bldg 434A, Argonne National Laboratory, 9700 South Cass Ave., Lemont, IL 60439, USA.

^4^ European XFEL GmbH, Holzkoppel 4, 22869 Schenefeld, Germany.

^5^ School of Biological Sciences, University of Aberdeen, Cruickshank Building, St Machar Drive, Aberdeen AB24 3UU, Scotland, UK.

^6^ Department of Biology, National and Kapodistrian University of Athens, Panepistimiopolis, Athens 157 84, Hellas, Greece.

^7^ Department of Chemistry and Biochemistry, San Diego State University, CA, 92182-1030 USA.

^8^ University of South Bohemia, Department of Experimental Plant Biology, Branišovská 31/1160, 370 05 České Budějovice, Czech Republic.

^9^ Marine Biodiscovery Centre, Department of Chemistry, University of Aberdeen, Aberdeen AB24 3UE, Scotland, UK.

^+^ This paper is dedicated to our friend Prof. Carl J. Carrano (14 July 1950–26 January 2022), who suddenly passed away during the writing of this paper. He will be remembered for his contributions to inorganic biochemistry, especially of marine organisms.

^#^ Author for correspondence: [fkuepper@abdn.ac.uk](mailto:fkuepper@abdn.ac.uk)

**X-ray microprobe images** (see Fig. S1) in 2009 yielded the first crude approach to elemental abundance and distribution in *E. siliculosus*. In particular, the concentrations of Br, Ca, Cl, Cu, Fe, I, K, Ni, Sr and Sr were found to be strongly increased compared to the background levels, while those of Ga and Mn showed no accumulation and that of S was just barely above the detection threshold. The iodine K line proved to be substantially more informative than the L line. These images prompted the further investigation of strontium accumulation in brown algae in the context of this study.

**Fig. S1.** X-ray microprobe maps feasibility test of elemental distribution in *E. siliculosus*. Element distribution of A) bromine (K line), B) calcium (K line), C) chlorine (K line), D) copper (K line), E) iron (K line), F) gallium (K line), G) iodine (K line), H) iodine (L line), I) potassium (K line), J) manganese (K line), K) nickel (K line), L) sulfur (K line), M) strontium (K line) and N) zinc (K line). Measurements were done in 2009 at beamline P06 (PETRA III synchrotron, Hamburg. Germany) equipped with a 2-m spectroscopy undulator. The first optical element is a cryogenically cooled double-crystal monochromator located at 38.5 m from the source. It holds pairs of Si (1 1 1) and (3 1 1) crystals and can cover an energy range from 2.4 to 100 keV, generating a fixed vertical offset of 21 mm from the storage ring plane. Microfocusing is done with a Kirkpatrick–Baez mirror system and by parabolic refractive X-ray lenses at lower and higher energies, respectively. Hard X-ray beam sizes down to the 300 nm level are to be used for scanning microscopy with fluorescence, absorption spectroscopic and diffraction contrast as described before [1]. For the present work, a bending magnet insertion device and a single-bounce capillary optics with a beam size of 10 microns were used. The whole set-up is described in Thomas et al. [2].


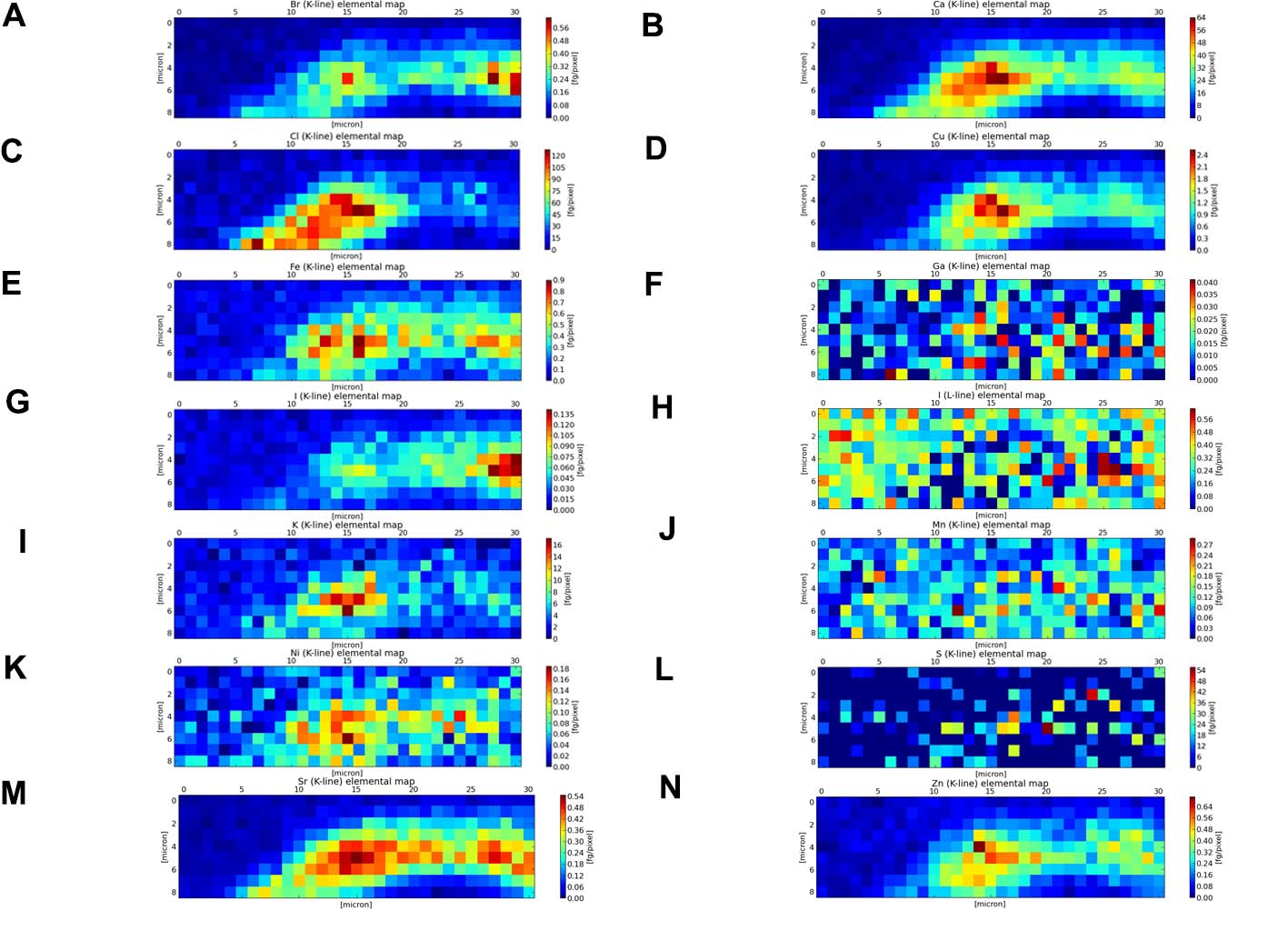


**Linear composition fit of *Laminaria digitata* and *Ectocarpus siliculosus***.

The spectra of *Laminaria digitata* cortex and meristoderm tissues, and the spectrum of *Ectocarpus siliculosus* whole blade tissue (bulk) from Böttger et al. were used for linear composition fit. For each spectrum a fit was done using the other two as components (see Fig S2 and Table S1) [3]. This analysis quantifies the similarity among the tissues (cortex vs meristoderm) and which form of Fe is mostly stored in the algae blade. The fit was done using ATHENA in a range of (-20,+80) eV energy range around the absorption edge [4].

The spectrum of *E. siliculosus* whole blade (bulk) sample CC47 was explained as a non -ferritin ferrihydrite mineral with a bigger particle size than the ferrihydrite core of ferritin [3]. The whole blade of *E. siliculosus* could be fitted by a linear combination of the cortex and meristoderm of *L. digitata*, with 72% of cortex in agreement with the cortex having the most Fe concentration (see Fig.1 and 3 in main text). Only 98% of the spectrum of *L. digitata* cortex and 72% of the meristoderm could be explained by the *E. siliculosus* spectrum, indicating that the meristoderm includes other form of Fe, while the cortex is mostly ferrihydrite mineral.

**
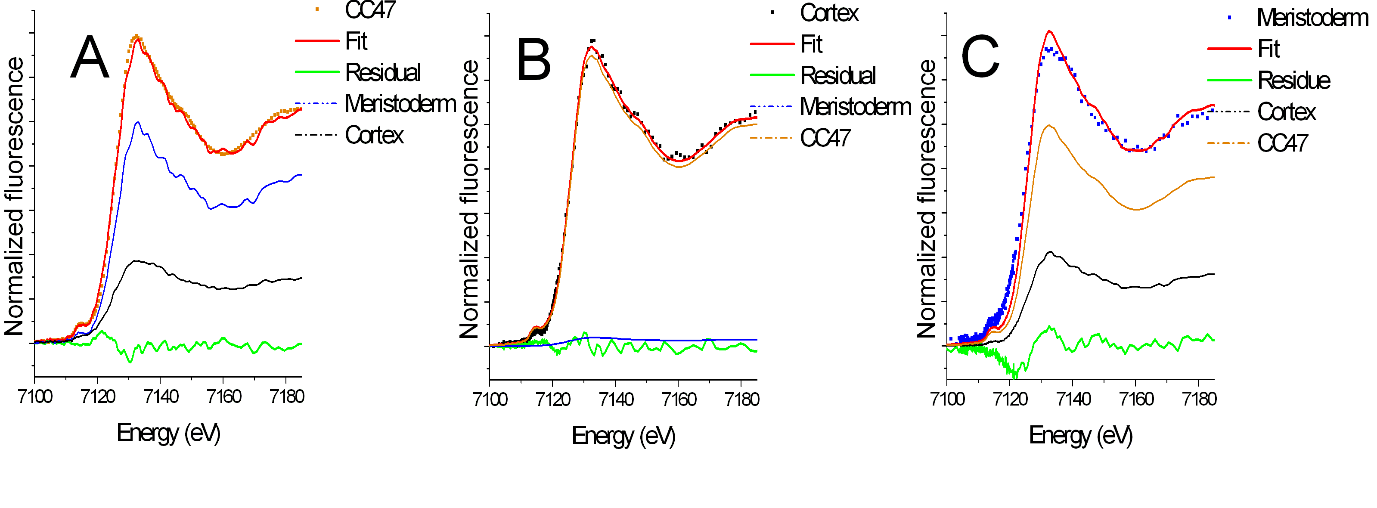
**

**Fig. S2.** Linear composition fit of: A*) Ectocarpus siliculosus* whole blade (bulk) sample CC47, and *Laminaria digitata,* B) cortex and C) meristoderm. Each sample was analyzed as linear composition of the other two spectra.

**Table S1. Composition and R factor for the linear composition fit of Fig. S2.**

| Sample | *E. siliculosus* bulk (CC47) % | *L. digitata* cortex % | *L. digitata* meristoderm % |
| --- | --- | --- | --- |
| *E. siliculosus* bulk (CC47) | - | 95 | 72 |
| *L. digitata* cortex | 72 | - | 31 |
| *L. digitata* meristoderm | 28 | 3 | - |
| Total sum | 100 | 98 | 103 |
| R factor | 0.002 | 0.0016 | 0.017 |

**Linear composition fit using references**

The linear composition was fitted for the absorption edge region in a range [-5, +45] eV using ATHENA. We have used the reference compounds ferrihydrite and FeIII-sulphate, that were digitized from the publication using Engauge Digitizer Software [5,6].

In all samples ferrihydrite is the only component that could be fitted. The meristoderm spectrum has low statistical quality due to the low Fe content, giving a bad quality fit. For the bulk sample of *E. siliculosus* (sample CC47) and the cortex of *L. digitata*, the main component appears to be a ferritin-like ferrihydrite core. The R factor values obtained are: 0.0496 for the bulk *E. siliculosus* (sample CC47), 0.0086 for the cortex and 0.065 for the meristoderm fit.


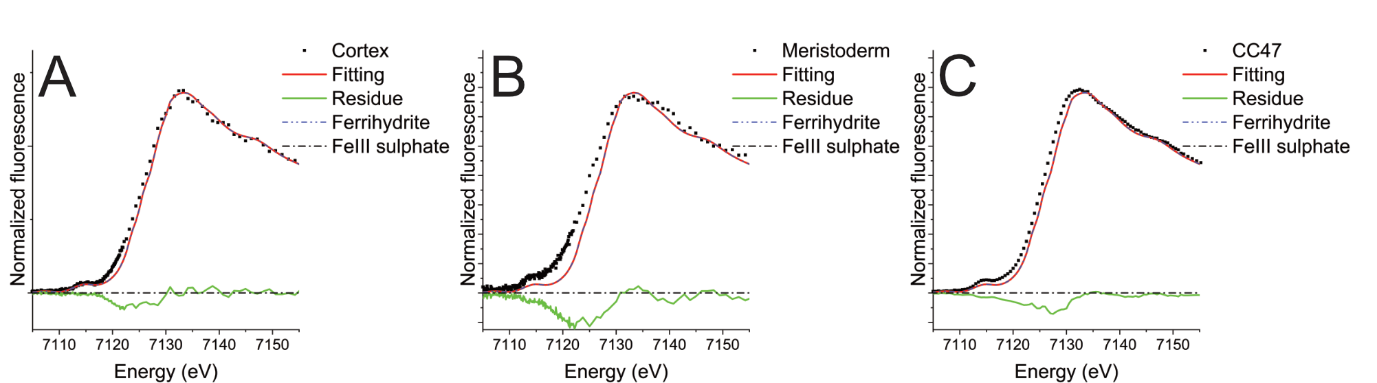


**Fig. S3.** Linear composition fit of: A*) Ectocarpus siliculosus* whole blade (bulk) sample CC47, and *Laminaria digitata,* B) cortex and C) meristoderm. Each sample was analyzed as linear composition of the ferrihydrite and FeIII-sulphate samples from Pattammattel et al, but only ferrihydrite could be fitted [6].

**
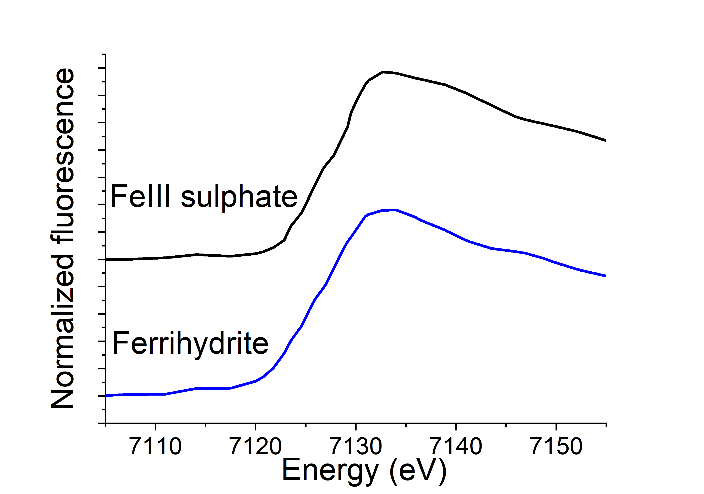
**

**Fig. S4.** References of Fe K-edge XANES of ferrihydrite and FeIII-sulphate digitized from Pattammattel et al [6].

**Colocalization of elements in the nano-tomography images**

Colocalization images (top) were done in MATLAB using the quantified RGB images shown in the main text, with primary channel: red for Fe/Zn, green for Sr. The structural similarity index map (SSIM) (bottom) compares luminance, contrast and structure between the Fe/Zn and Sr (highest similarity at top of colorbar in red color) [7]. Panel figures were assembled in Affinity Designer. *L. digitata* is shown in Fig. S5 and *E. siliculosus* in Fig. S6.


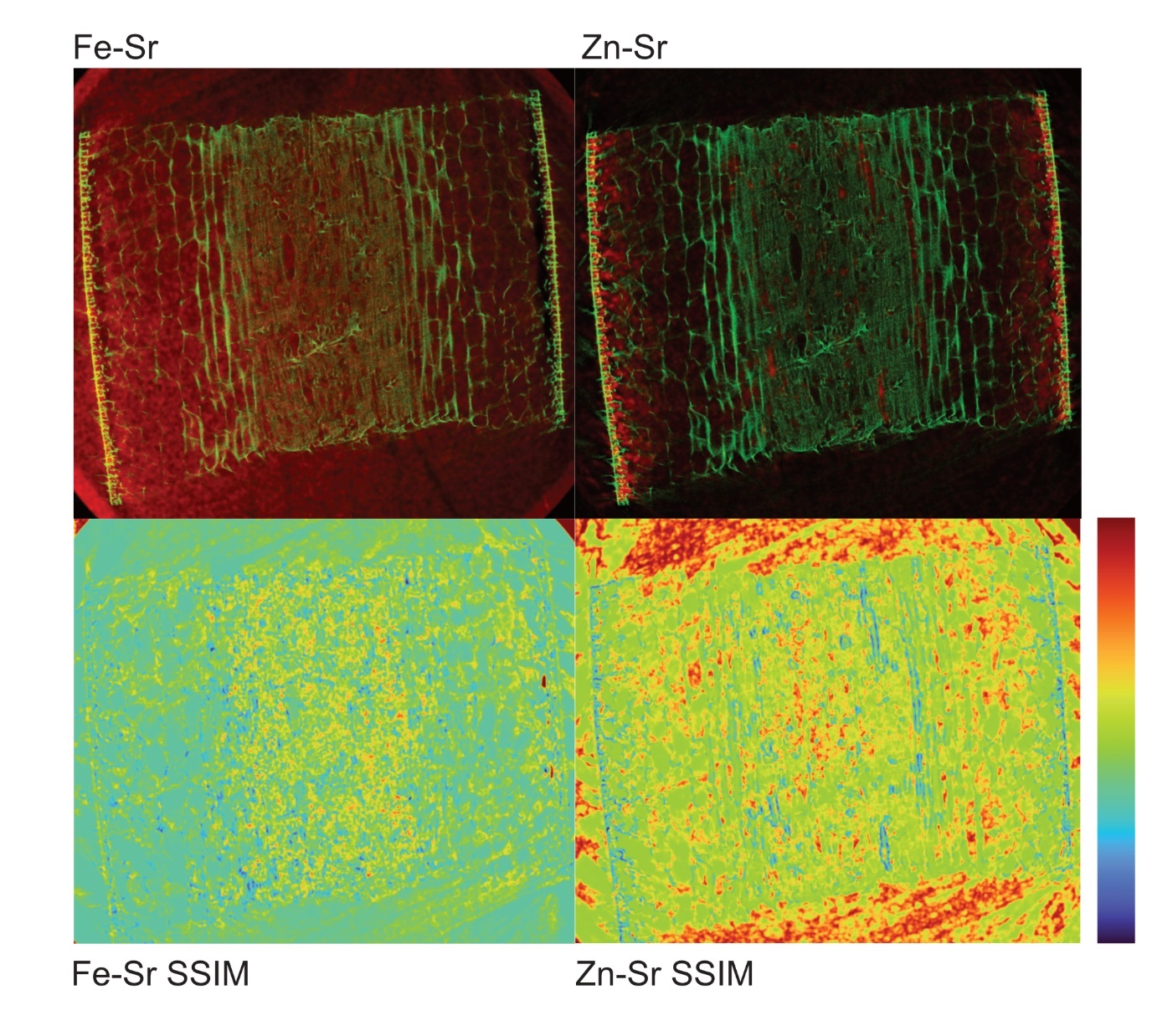


**Fig. S5.** Colocalization of Fe (red) and Sr (green), and Zn (red) and Sr (green) for *L. digitata* sample **LD10,** showing the whole tissue measured and part of the capillary (circle sections, 1 mm diameter) used for the nano-tomography in beamline 16A at ESRF. At bottom, the structural similarity index maps (SSIM) show the areas of colocalization of Fe/Zn and Sr (highest similarity at top of colorbar in red color) [7].

**
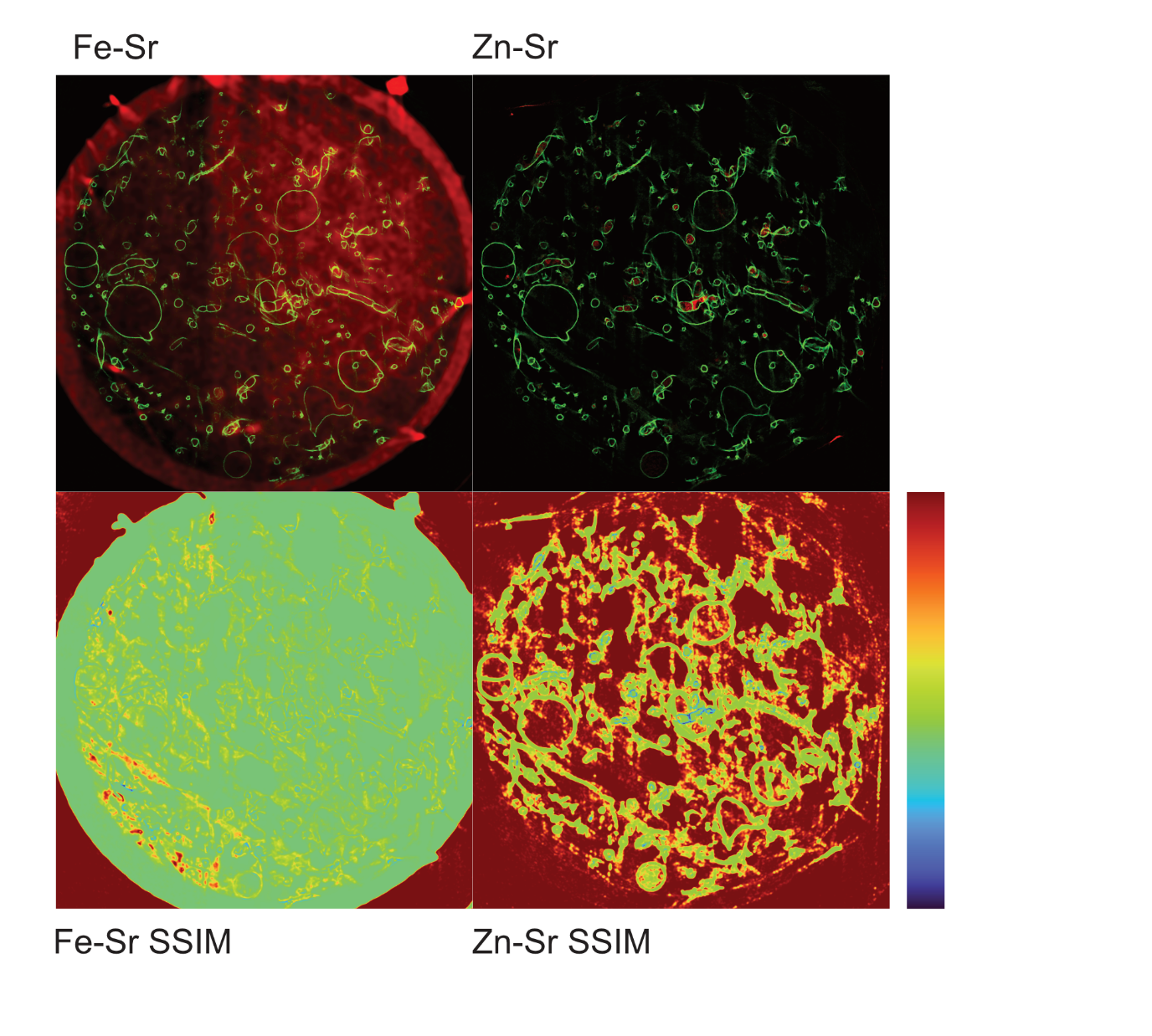
**

**Fig. S6.** Colocalization of Fe (red) and Sr (green), and Zn (red) and Sr (green) for *E. siliculosus***,** showing the whole tissue measured and part of the capillary (circle sections, 1 mm diameter) used for the nano-tomography in beamline 16A at ESRF. At bottom, the structural similarity index maps (SSIM) show the areas of colocalization of Fe/Zn and Sr (highest similarity at top of colorbar in red color) [7].

**Sr localization in L. digitata**

The Sr fluorescence nano-tomography of sample **LD2** of *L. digitata* (green) was colocalized manually and subtracted from the X-ray phase contrasting tomography(magenta) using ImageJ/Fiji. Dark areas show region of colocalization of Sr with high density areas of the hologram (higher concentrations/densities in lighter color) (see Fig. S7).

**
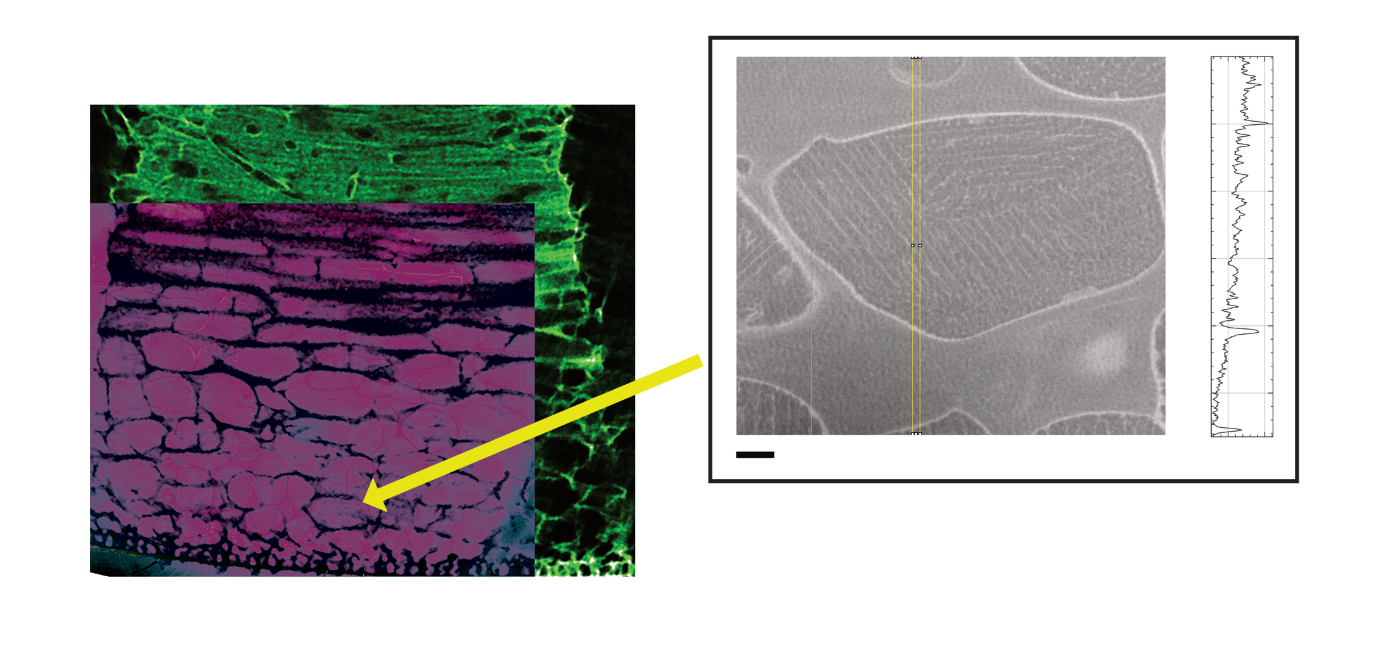
**

**Fig. S7.** Difference of X-ray phase contrasting tomography (magenta, high density areas in light hue) and the Sr fluorescence nano-tomography of sample **LD2** of *L. digitata* (high concentration in green light hue): the dark areas show the colocalization of the high-density areas in the hologram and the Sr localization in the fluorescence image. It shows that Sr is indeed located at the plasmalemma membrane. Zoom figure: X-ray phase contrasting tomography collected at beamline 16A at ESRF on sample **LD2** of *L. digitata*. The intensity of the profile (yellow) is shown on the right. The high-density area is in light gray and is compatible with the plasmalemma membrane. Scale bar = 7 µm.

**Transcriptomics**

The Ferritin-like superfamily of diiron-containing four-helix-bundle proteins are (<https://www.ncbi.nlm.nih.gov/Structure/cdd/cddsrv.cgi?uid=412271>):

1. acsF phytochrome-regulated gene [ Fucus vesiculosus ] Gene ID: 11542210 <https://www.ncbi.nlm.nih.gov/gene/11542210>
2. acsF [ Sargassum fusiforme ]Gene ID: 55291802 <https://www.ncbi.nlm.nih.gov/gene/55291802> (YP_009828267.1)
3. acsF [ Sargassum horneri ] Gene ID: 27219246 <https://www.ncbi.nlm.nih.gov/gene/27219246> (YP_009243720.1)
4. acsF [Sargassum thunbergii] Gene ID: 26830896

<https://www.ncbi.nlm.nih.gov/gene/26830896> (YP_009227344.1)

Table S2. BLAST search results for ferritin orthologs.

| Description | Scientific name | Max score | Total score | Query cover | E value | Per. Ident | Acc. Len | Accession | Searched protein query |
| --- | --- | --- | --- | --- | --- | --- | --- | --- | --- |
| Phytochrome-regulated protein | *Undaria pinnatifida* | 581 | 581 | 97% | 0 | 75.9 | 366 | YP_009182527.1 | *F. vesiculosus* ferritin acsF gene product |
|  |  | 565 | 565 | 96% | 0 | 72.91% | 366 | YP_009182527.1 | *S. fusiforme* ferritin acsF |
|  |  | 567 | 567 | 96% | 0 | 72.91% | 366 | YP_009182527.1 | *S. horneri* ferritin acsF |
|  |  | 566 | 566 | 96% | 0 | 73.18% | 366 | YP_009182527.1 | *S. thunbergii* ferritin acsF |
| Phytochrome-regulated protein | *Saccharina japonica* | 574 | 574 | 97% | 0 | 74.52% | 366 | YP_006639047.1 | *F. vesiculosus* ferritn acsF gene product |
|  |  | 563 | 563 | 96% | 0 | 72.63% | 366 | YP_006639047.1 | *S. fusiforme* ferritin acsF |
|  |  | 564 | 564 | 96% | 0 | 72.35% | 366 | YP_006639047.1 | *S. horneri* ferritin acsF |
|  |  | 564 | 564 | 96% | 0 | 72.35% | 366 | YP_006639047.1 | *S. horneri* ferritin acsF |
|  |  | 566 | 566 | 96% | 0 | 72.91% | 366 | YP_006639047.1 | *S. thunbergii* ferritin acsF |
| Magnesium-protoporphyrin ix monomethyl ester (oxidative) cyclase | *Ectocarpus siliculosus* | 560 | 560 | 96% | 0 | 73.11% | 365 | YP_003289215.1 | *S. fusiforme* ferritin acsF |
|  |  | 560 | 560 | 96% | 0 | 72.83% | 365 | YP_003289215.1 | *S. horneri* ferritin acsF |
|  |  | 561 | 561 | 96% | 0 | 73.11% | 365 | YP_003289215.1 | *S. thunbergii* ferritin acsF |
|  |  | 567 | 567 | 98% | 0 | 73.55% | 365 | YP_003289215.1 | *F. vesiculosus* ferritin acsF gene product |

**References**

1. Schroer CG, Boye P, Feldkamp JM, Patommel J, Samberg D, Schropp A, Schwab A, Stephan S, Falkenberg G, Wellenreuther G, Reimers N. Hard X-ray nanoprobe at beamline P06 at PETRA III. *X-Ray Mirror* 2010;**616**:93–7.

2. Thomas G, Stärk H-J, Wellenreuther G, Dickinson BC, Küpper H. Effects of nanomolar copper on water plants—Comparison of biochemical and biophysical mechanisms of deficiency and sublethal toxicity under environmentally relevant conditions. *Aquat Toxicol* 2013;**140–141**:27–36.

3. Böttger LH, Miller EP, Andresen C, Matzanke BF, Küpper FC, Carrano CJ. Atypical iron storage in marine brown algae: a multidisciplinary study of iron transport and storage in Ectocarpus siliculosus. *J Exp Bot* 2012;**63**:5763–72.

4. Ravel B, Newville M. ATHENA, ARTEMIS, HEPHAESTUS: data analysis for X-ray absorption spectroscopy using IFEFFIT. *J Synchrotron Radiat* 2005;**12**:537–41.

5. Mitchell M, Muftakhidinov B, Winchen T, Wilms A, Schaik BV, Badshah400, Mo-Gul, Badger TG, Jędrzejewski-Szmek Z, Kensington, Kylesower. Engauge Digitizer Software. 2020, DOI: 10.5281/ZENODO.3941227.

6. Pattammattel A, Tappero R, Gavrilov D, Zhang H, Aronstein P, Forman HJ, O’Day PA, Yan H, Chu YS. Multimodal X-ray nano-spectromicroscopy analysis of chemically heterogeneous systems. *Metallomics* 2022;**14**, DOI: 10.1093/mtomcs/mfac078.

7. Wang Z, Bovik AC, Sheikh HR, Simoncelli EP. Image quality assessment: from error visibility to structural similarity. *IEEE Trans Image Process* 2004;**13**:600–12.
